# Different Patterns of Attention Modulation in Early N140 and Late P300 sERPs Following Ipsilateral vs. Contralateral Stimulation at the Fingers and Cheeks

**DOI:** 10.3389/fnhum.2021.781778

**Published:** 2021-12-06

**Authors:** Laura Lindenbaum, Sebastian Zehe, Jan Anlauff, Thomas Hermann, Johanna Maria Kissler

**Affiliations:** ^1^Department of Psychology, Bielefeld University, Bielefeld, Germany; ^2^Center for Cognitive Interaction Technology (CITEC), Bielefeld University, Bielefeld, Germany; ^3^Faculty of Technology, Bielefeld University, Bielefeld, Germany

**Keywords:** somatosensory ERPs, N140, P300, hemisphere, somatosensory representation, stimulus manipulation, EEG

## Abstract

Intra-hemispheric interference has been often observed when body parts with neighboring representations within the same hemisphere are stimulated. However, patterns of interference in early and late somatosensory processing stages due to the stimulation of different body parts have not been explored. Here, we explore functional similarities and differences between attention modulation of the somatosensory N140 and P300 elicited at the fingers vs. cheeks. In an active oddball paradigm, 22 participants received vibrotactile intensity deviant stimulation either ipsilateral (within-hemisphere) or contralateral (between-hemisphere) at the fingers or cheeks. The ipsilateral deviant always covered a larger area of skin than the contralateral deviant. Overall, both N140 and P300 amplitudes were higher following stimulation at the cheek and N140 topographies differed between fingers and cheek stimulation. For the N140, results showed higher deviant ERP amplitudes following contralateral than ipsilateral stimulation, regardless of the stimulated body part. N140 peak latency differed between stimulated body parts with shorter latencies for the stimulation at the fingers. Regarding P300 amplitudes, contralateral deviant stimulation at the fingers replicated the N140 pattern, showing higher responses and shorter latencies than ipsilateral stimulation at the fingers. For the stimulation at the cheeks, ipsilateral deviants elicited higher P300 amplitudes and longer latencies than contralateral ones. These findings indicate that at the fingers ipsilateral deviant stimulation leads to intra-hemispheric interference, with significantly smaller ERP amplitudes than in contralateral stimulation, both at early and late processing stages. By contrast, at the cheeks, intra-hemispheric interference is selective for early processing stages. Therefore, the mechanisms of intra-hemispheric processing differ from inter-hemispheric ones and the pattern of intra-hemispheric interference in early and late processing stages is body-part specific.

## Introduction

Event-Related Potentials (ERPs) are widely explored in visual and auditory oddball designs, but commonly examined brain responses occur also in somatosensory oddball stimulations (Desmedt and Robertson, 1977; Yamaguchi and Knight, 1991; Satomi et al., 1995; Ito and Takamatsu, 1997; Nakajima and Imamura, 2000a). The functional significance of various scalp potentials evoked by sensory stimuli has been extensively examined, often in oddball paradigms. Some such potentials, which can be identified by their polarity and latency, are for example the N100, N200, P200, and P300. The N100 ERP reflects the sensory processing of the stimuli (Näätänen and Picton, 1987). The function of the N200/P200 complex lies in the detection of differences in the sensory environment and is independent of the sensory modality of ongoing stimulus and the underlying neural processes can be similar to those of the cognitive P300 (Mouraux and Iannetti, 2009) which occurs in discrimination tasks, or oddball paradigms.

An early attention-sensitive prefrontal component is the tactile N140. It is functionally analogous to the auditory and visual N100 component. The N140 is evoked at a latency of about 140 ms after stimulus onset (Desmedt and Robertson, 1977) and generated bilaterally in distributed regions of the frontal lobes (Allison et al., 1992). Like other N1 responses, the somatosensory N140 is larger for attended than for unattended stimuli (Desmedt and Robertson, 1977; Garcia-Larrea et al., 1995; Kida et al., 2004a). An important later ERP in oddball stimulation is the centro-parietal P300, as discussed further, a positive deflection in averaged EEG data that arises 300 ms to 500 ms after the target stimulus onset as a response to deviant stimuli. Despite differences in the latency and amplitude between the auditory, visual, and somatosensory stimulus modalities, the scalp topography of the P300 is broadly similar in all three modalities (Snyder et al., 1980).

Even though somatosensory ERPs are not as widely explored as those from other modalities, their amplitudes and latencies are known to be affected by manipulations of stimulus intensity, task difficulty, duration, and inter-stimulus interval (ISI) as well as stimulated body location and its respective somatosensory representation (Pfefferbaum et al., 1984; Tomberg et al., 1989; Nakajima and Imamura, 2000a, b; Spackman et al., 2007; Wang et al., 2008; Severens et al., 2010; Shen et al., 2018). Nakajima and Imamura (2000a) demonstrated that both somatosensory N140 and P300 deflections in response to deviant stimuli increase as a function of stimulus intensity. Results showed higher N140 and P300 deflections with increasing stimulus intensity in the active as well as in the passive oddball paradigm. Nakajima and Imamura (2000b) further showed that the P300 amplitude increases with longer ISI.

With regard to stimulated body locations, Shen et al. (2018) revealed that, at least within one hemisphere, the distance between the cortical representations of the stimulated body parts has an effect on early automatic somatosensory mismatch responses elicited in passive oddball paradigms, whereas the P300 is affected by the distance between these body parts on the body surface. They reported that stimulating locations with a bigger distance between the cortical representations, such as lip and neck compared with lip and hand, elicited significantly higher amplitudes and shorter latencies in early automatic somatosensory mismatch response. The mismatch negativity (MMN) is a frontocentrally distributed ERP, similar to the N1, which occurs as a response to an auditory as well as somatosensory deviant stimulus using a passive oddball paradigm (Campbell et al., 2007; Shen et al., 2018). For the P300, on the other hand, Shen and colleagues showed significantly higher amplitude in the stimulation of the index and fifth finger than in the stimulation of the index and third finger and also a significantly higher P300 response to the more distant lip/hand contrast than to the closer lip/neck contrast. Although they used a passive paradigm, they could show fundamental effects on the amplitude as well as the latency of somatosensory ERPs by stimulating different body locations. Thus, at least for within-hemisphere stimulation, amplitude of the early mismatch responses appeared sensitive to the distance between cortical representations of body parts, whereas P300 was more sensitive to the distance on the body surface.

However, multiple studies showed that when stimulating body parts with neighboring cortical representation within one hemisphere, these representations interact, reflecting intra-hemispheric interference.

Biermann et al. (1998) showed in a magnetoencephalography study examining somatosensory evoked fields elicited by individual finger stimulation that simultaneous stimulation of adjacent digits resulted in stronger inhibitory interaction than the simultaneous stimulation of non-adjacent digits. Also, Pang and Mueller (2015) and Severens et al. (2010) showed significantly smaller steady-state somatosensory evoked potentials (SSSEPs) for stimulation within a hand vs. stimulation of fingers on different hands. Pang and Mueller (2015) created an experiment in which participants had to focus on either one or both stimuli in a within-hand and a between-hands condition. In the within-hand condition, participants had to focus on either the index finger, ring finger, or both fingers. In the between-hands condition, participants had to focus on either the left ring finger, right ring finger, or both. Their electrophysiological results showed only intra-hemispheric interference, with significantly smaller SSSEPs for the within-hand condition, but no interference between hemispheres, although events were significantly more salient with longer duration in the within-hand paradigm, likely reflecting greater competitive interactions for within-hands compared to the between-hands stimulation. Furthermore, Breitwieser et al. (2011) showed that classifying SSSEPs on one finger against a reference period without focused attention is successfully possible whereas discriminating *via* machine learning classifiers between SSSEPs elicited from different fingers on the same hand is more challenging. These findings indicate that excitatory and inhibitory synaptic connections may overlap and interfere with each other when two stimulated areas have neighboring representations (Biermann et al., 1998; Hoechstetter et al., 2001). This interference may be reduced by increasing the cortical distance of stimulated areas (Mountcastle, 1984).

Moreover, interference may occur in specific time windows and may be differently affected by cortical distance vs. distance on the body surface, with early ERPs being sensitive to distance of cortical representations whereas late ERPs reflecting distance on the body (Shen et al., 2018). Finally, mechanisms of intra-hemispheric stimulation may differ from inter-hemispheric ones.

In the current study, we aimed to expand knowledge on mechanisms of attention modulation of somatosensory processing and examined responses to somatosensory deviant stimulation at different body parts (cheeks and fingers), as well ipsilateral (within-hemisphere) and contralateral (between-hemisphere) stimulation in an active oddball paradigm, because the active oddball paradigm leads to stronger responses than passive paradigms (Näätänen, 1992).

We analyzed ERPs in response to discrete events rather than continuous oscillatory stimulation such as the above-mentioned SSSEPs, which arguably represent at least partly a continuous sequence of overlapping N1 responses (Müller and Hillyard, 2000). Thereby, we addressed the temporal sequence of brain responses time-locked to the eliciting stimulus and focused on the N140 and P300 components. Thus, we combined the ERP method of Shen et al. (2018) with an active attention task as used in Pang and Mueller (2015). We aimed to investigate functional similarities and differences among the N140 and P300 measures of somatosensory processing and wanted to explore to what extent any effects are specific to the representation of certain body parts (fingers vs. face). Since stimulation at adjacent fingers on a single hand is assumed to cause interference, we focused on increasing the distance between the stimulated areas (contralateral). We also covered a larger area of the skin in ipsilateral deviant stimulation following the study of Pang and Mueller (2015), hypothesizing that simultaneous ipsilateral stimulation of three fingers would cause interference due to the neighboring finger representation in the somatosensory cortex. We tested the effects of the ipsilateral stimulation at the cheek, when stimulating an area of the same spatial extent as at the fingers.

For the earlier N140 component, we hypothesized higher amplitudes and shorter latencies in the contralateral one finger deviant stimulation, compared to the ipsilateral condition, where index, middle, and ring finger were simultaneously stimulated, as we anticipated intra-hemispheric interference in ipsilateral deviant stimulation. We tested, whether the same would be true for stimulation at the cheek when a larger ipsilateral area would be stimulated, compared to a smaller contralateral area. Extending the idea of Shen et al. (2018) to processing across the two hemispheres, to increase the distance between the cortical representations of the stimulated body part, we hypothesized that the N140 amplitude is more negative and latency shorter for the contralateral deviant stimulation at the cheek than the contralateral deviant presentation at the fingers, since the cheeks are represented more laterally than the fingers, yielding largest distance between the cheek representations. For the later P300 component, we hypothesized that the contralateral stimulation at the finger causes higher P300 amplitudes and shorter latencies than the ipsilateral stimulation at the fingers. Also, since, at least in intra-hemispheric processing, the P300 has been reported to be affected by the distance between stimulated areas on the body surface we hypothesized that the contralateral stimulation at the finger would elicit higher amplitudes and shorter latencies than the contralateral stimulation at the cheeks.

## Materials and Methods

### Participants

Twenty-two participants (17 females) took part in this study, yielding a comparable sample size and gender distribution as other recent publications in the area (Nakajima and Imamura, 2000b; Pang and Mueller, 2015; Shen et al., 2018). Each of them received 10€ or course credits for participation and gave informed consent to participate in this study. All of them were right-handed (ø 98,79 Edinburgh Handedness Inventory) and they were 25.45 years on average (SD = 3.17). Neither of them reported a neurological or psychological disorder nor the intake of medications or drug abuse. The research was approved by the ethics committee of the German Psychological Association.

### Stimulation System and Experimental Paradigm

**Figure 1:**
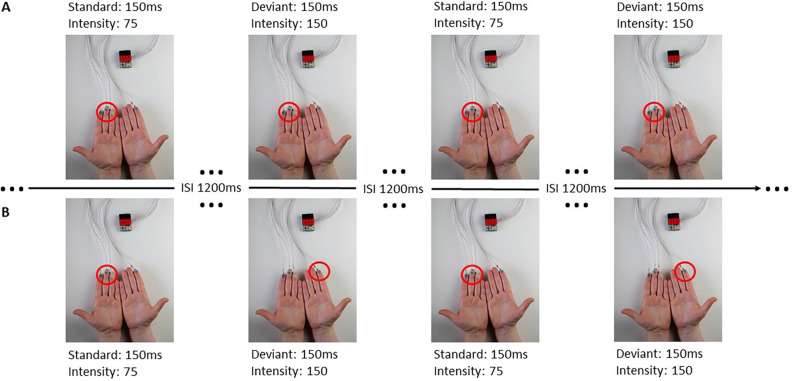
BRIX_2_ with extension module and experimental procedure. Panel **(A)** represents a block with all stimuli presented ipsilaterally at the left fingers, the red circle indicating the activated stimulation site. Panel **(B)** demonstrates a contralateral block with standard stimuli presented at the left fingers and deviant stimuli presented at the right index. The sequence of standard and deviant stimuli in this Figure is an example. Each block consisted of 200 standard and 40 deviant stimuli.

The stimuli were presented through the BRIX_2_ prototyping system, developed inhouse (Zehe, 2018). The base-module of the BRIX_2_ consists of an Arduino compatible microcontroller, a 9-DOF IMU (inertial measurement unit), a wireless interface, and a LiPoly-battery. BRIX_2_ is a light but robust system, which allows the user to attach extension modules through three extension headers on the top of the base-module, packaged in LEGO bricks. In this study, we used an extension-module with cell-type-vibration motors (ERM; 10 mm × 3 mm; see Figure 1).

The base-module firmware is an Arduino sketch allowing control of each vibration motor separately. The amplitude of the vibrations can be modified in the sketch and allows the user to choose a value between 0 and 255 where 0 is no vibration at all. These values can be normalized with 255 as the maximum vibration of the motors (100%). The duration of the vibration can also be defined in the sketch. To control the application, the base-module was connected to the stimulation computer (DELL Latitude D830) *via* USB, deviant and standard stimuli were then triggered through commands over a serial interface. A fixed stimulus sequence was created for each block in OpenSesame, but the order of the experimental blocks was randomized for each participant (see Table 1).

**Table 1:** Experimental setup and spots where the stimuli were presented.

Laterality of deviant presentation	Body part (standard)	Body part (deviant)
ipsilateral	left fingers	left fingers
ipsilateral	right fingers	right fingers
ipsilateral	left cheek	left cheek
ipsilateral	right cheek	right cheek
contralateral	left fingers	right index finger
contralateral	right fingers	left index finger
contralateral	left cheek	right cheek
contralateral	right cheek	left cheek

### Stimuli

Each stimulus had a duration of 150 ms. Standard stimuli had an intensity value of 75 (29.41% of the maximum vibration, magnitude 0.33 *g*) and a peak frequency of 92.5 Hz, the deviant stimulus had an intensity value of 150 (58.85% of the maximum vibration, magnitude 0.8 *g*), and a peak frequency of 175 Hz. The inter-stimulus-interval (ISI) was 1,200 ms and the ratio of standards to deviants was 5:1, whereby no two deviant stimuli followed consecutively.

For the ipsilateral stimulation, standard and deviant stimuli were always presented at the same spot of the body. Therefore, three motors were attached to the fingers (index, middle, and ring finger), (see Figure 1) or at boneless parts at the cheeks. For the contralateral stimulation, a single deviant stimulus was presented at the index finger (see Figure 1) or at the cheek on the opposite side (see Table 1). All stimulations were performed on both sides of the body (right and left) and were later averaged across left and right stimulation sites, separately for each body part.

### EEG Recording and Preprocessing

The Electroencephalography signals were recorded using a BioSemi system with 128 active electrodes^1^ and a sampling rate of 1,024 Hz. The data was recorded with the Cz reference and off-line re-referenced to the average reference. Data were pre-processed with BESA^®^ Research 6.0 using a high-pass filter of 0.16 Hz, a low-pass filter of 30 Hz, and the automatic artifact detection implemented in the software. In total 0.178 percent of all electrode measurements were interpolated due to artifacts and noisy data. Data were segmented into epochs from 100 ms before stimulus onset to 800 ms after stimulus onset, the 100 ms before stimulus onset serving as the baseline. The data were averaged per condition and participant and saved for further analysis in MATLAB^®^ and EMEGS (Peyk et al., 2011).

### Procedure

The participants were informed about the study and its general background and gave informed consent. They had to fill out a questionnaire regarding demographics, medical, and psychological health, intake of medication or drug abuse as well as the Edinburgh Handedness Inventory. Hereafter, the EEG cap was put on and the vibration motors were placed on the first locations. We defined eight blocks of how and where the participants were stimulated (see Table 1). In four of the blocks, stimulation was presented ipsilaterally which means that the standard and deviant stimuli were presented at the same spot of the body. In the other four blocks stimulation was presented contralaterally, where standard stimuli were presented on one side of the body with three motors (e.g., right fingers) and the deviant stimuli were presented on the contralateral side with a single motor (e.g., single deviant on left index finger).

Adhesive plasters were used to attach the motors. Each block consisted of 200 standard and 40 deviant stimuli. Participants sat in a comfortable armchair and were instructed to sit in their favorite sitting position. During the oddball paradigm, participants had to focus on the deviant stimuli and count their occurrences.

### Data Analyses

**Figure 2:**
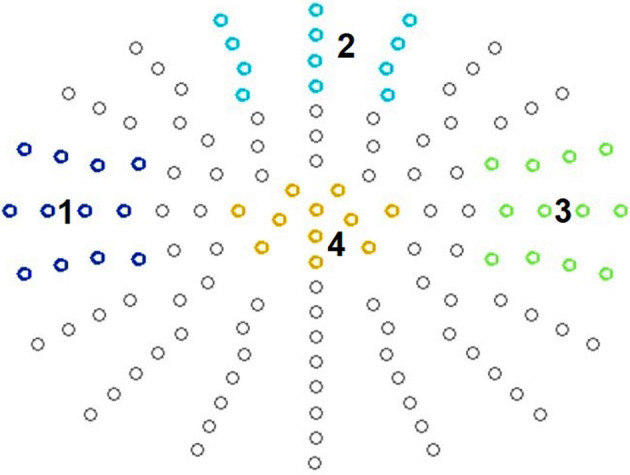
Defined clusters. N140: temporal left (1), frontal cluster (2), and temporal right (3). P300: centro-parietal cluster (4).

For ERP data analysis, we defined four clusters (see Figure 2) and averaged data across these clusters of interest. This by now common approach for statistical analysis guards against inflation of Type I error rate (Luck and Gaspelin, 2017) and at the same time can also improve data quality (Luck, 2014).

#### N140 Analysis

It is known that somatosensory stimulation leads to early fronto-central as well as temporal scalp distributions contralateral to the stimulation side in both passive and active oddball paradigms (Garcia-Larrea et al., 1995; Kida et al., 2004a, b). Therefore, for the N140 analysis, we defined a fronto-central, a right temporal, and a left temporal electrode cluster, each consisting of 12 electrodes (Figure 2). This also corresponded to the empirically observed topography.

For the amplitude analysis, the most negative peak in each condition was identified in a time-window of 100 ms to 180 ms at the selected electrodes and then averaged across electrodes for each participant *via* in-house MATLAB^®^-based software. For amplitude visualization, all conditions were averaged in EMEGS. The N140 peak latency was defined as latency of the most negative peak between 100 ms and 180 ms at each electrode of the electrode cluster. The latency was averaged across the electrodes in the N140 cluster for each participant.

#### P300 Analysis

For the P300 component, a central electrode cluster was defined consisting of 11 electrodes, corresponding to the typically observed P300 topography (Figure 2). For amplitude analysis the most positive peaks in all conditions were identified in a time-window of 250 ms to 550 ms at the selected electrodes and then divided by the number of electrodes for each participant *via* in house MATLAB^®^-based software. For amplitude visualization all conditions were averaged in EMEGS. The P300 peak latency was defined as latency of the most positive peak between 250 ms and 550 ms at all electrodes of the electrode cluster. Then latency was averaged across electrodes for each participant.

### Statistical Analysis

For the N140 component repeated measures ANOVAs with four factors (laterality of stimulation: ipsi- and contralateral; body part: fingers and cheek; stimuli: standard and deviant; and cluster group: left, frontal, and right) were set up to investigate the main effects and their interactions regarding amplitude and peak latency. Greenhouse-Geisser sphericity correction was only performed on the statistical analyses of the N140 since P300 analysis did not involve the factor electrode cluster. For the P300 component, repeated measures ANOVAs with three factors (laterality of stimulation, body part, and stimuli) were set up to investigate main effects and their interactions for amplitude and peak latency. Bonferroni corrections were used in all *post hoc* comparisons for the N140 and P300 components. Effect sizes are reported as partial eta-squared or Cohen’s d as appropriate.

## Results

### N140 Component (100–180 ms)

#### N140 Amplitude

**Figure 3:**
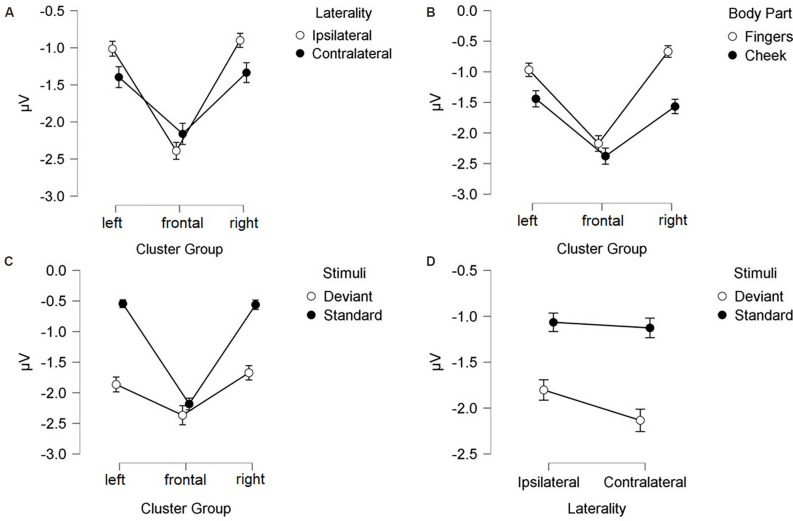
Mean values (μV) of N140 amplitudes and SE. Two-way-interactions: **(A)** Cluster Group x Laterality, **(B)** Cluster Group x Body Part, **(C)** Cluster Group x Stimuli, and **(D)** Laterality x Stimuli. SE, Standard Errors.

**Figure 4:**
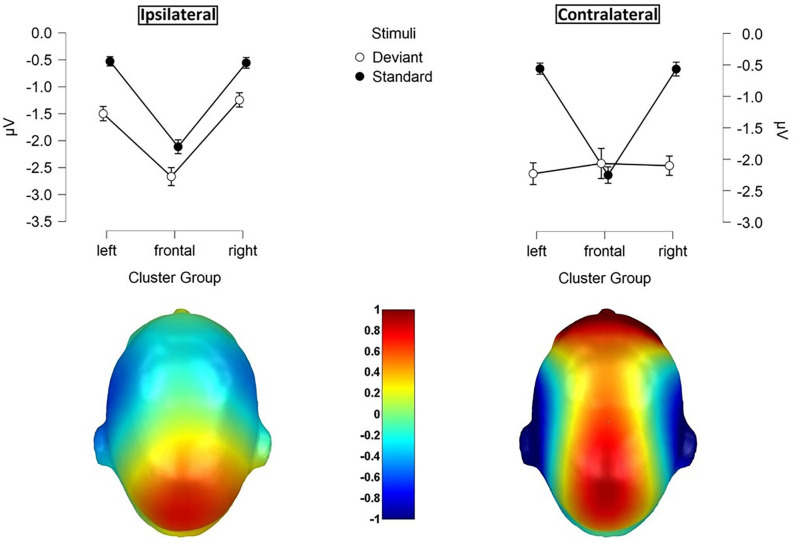
Mean values (μV) of N140 amplitudes and SE. Illustration of the three-way-interaction: Laterality x Cluster Group x Stimuli, with topographic difference plots (100–180ms). Bottom left: Ipsilateral Deviants—Ipsilateral Standards. Bottom right: Contralateral Deviants—Contralateral Standards.

N140 results are depicted in Figures 3, 4. Mean amplitudes for each condition are further summarized in Table 2. For the N140 Amplitude main-effects for stimuli (*F*_(1, 21)_ = 123.545; *p* < 0.001; *η*^2^ = 0.855), laterality (*F*_(1, 21)_ = 7.055; *p* = 0.015; *η*^2^ = 0.251), body part (*F*_(1, 21)_ = 48.100; *p* < 0.001; *η*^2^ = 0.696), and cluster group were found (*F*_(1.39, 29.18)_ = 23.194; *p* < 0.001; *η*^2^ = 0.525). In addition, a two-way-interaction between laterality and cluster group (*F*_(1.74, 36.62)_ = 15.095; *p* < 0.001; *η*^2^ = 0.418), which is shown in Figure 3A, indicated that contralateral stimulation caused higher amplitudes in the lateral left- (*p* = 0.011; *d* = 0.466) and right-cluster (*p* = 0.002; *d* = 0.529) than ipsilateral stimulation. A further two-way-interaction between cluster group and body part (*F*_(1.29, 27.19)_ = 5.036; *p* = 0.025; *η*^2^ = 0.193, Figure 3B) was due to the fact that stimuli presented at the cheek elicited more negative amplitudes than stimuli presented at the fingers in the lateral left- (*p* = 0.033; *d* = 0.404) and right-cluster (*p* < 0.001; *d* = 0.768), but not in the frontal-cluster. Moreover, the interaction between cluster group and stimuli (*F*_(1.54, 32.24)_ = 15.956; *p* < 0.001; *η*^2^ = 0.432, Figure 3C) arose, because deviant stimuli elicited higher amplitudes in the lateral left- (*p* < 0.001; *d* = −1.132) and right-cluster (*p* < 0.001; *d* = −0.954) than standard stimuli. There was no difference between responses to standard and deviant stimuli in the fronto-central cluster. Another two-way-interaction between laterality and stimuli (*F*_(1, 21)_ = 4.967; *p* = 0.037; *η*^2^ = 0.191) was found (Figure 3D).

**Table 2:** Mean amplitudes of N140 with SE values.

Laterality of deviant	Body part	Stimuli	Cluster group	Mean (μV)	SE
Ipsilateral	Fingers	Deviant	Left	–1.18	± 0.14
			Frontal	–2.54	± 0.25
			Right	–0.74	± 0.14
		Standard	Left	–0.35	± 0.09
			Frontal	–2.04	± 0.22
			Right	–0.21	± 0.09
	Cheek	Deviant	Left	–1.82	± 0.21
			Frontal	–2.79	± 0.25
			Right	–1.75	± 0.21
		Standard	Left	–0.70	± 0.13
			Frontal	–2.18	± 0.21
			Right	–0.91	± 0.11
Contralateral	Fingers	Deviant	Left	–2.00	± 0.23
			Frontal	–2.04	± 0.37
			Right	–1.60	± 0.19
		Standard	Left	–0.34	± 0.07
			Frontal	–2.07	± 0.23
			Right	–0.12	± 0.09
	Cheek	Deviant	Left	–2.47	± 0.31
			Frontal	–2.10	± 0.31
			Right	–2.60	± 0.28
		Standard	Left	–0.78	± 0.12
			Frontal	–2.44	± 0.20
			Right	–1.01	± 0.15

Contralateral deviants were more negative than ipsilateral deviants (*p* = 0.008; *d* = 0.539), ipsilateral standards (*p* < 0.001; *d* = 1.516) and contralateral standards (*p* < 0.001; *d* = −1.597). ERPs elicited by standards did not differ between the ipsi- and contralateral deviant condition. Moreover, a three-way-interaction between laterality, cluster and stimuli was found (*F*_(1.74, 36.57)_ = 16.536; *p* < 0.001; *η*^2^ = 0.441; Figure 4).

ERPs to standard stimuli, presented ipsi- as well as contralaterally, were higher in the frontal-cluster than in the lateral left- (ipsilateral: *p* < 0.001; *d* = 0.674 | contralateral: *p* < 0.001; *d* = 0.720) and right-cluster (ipsilateral: *p* < 0.001; *d* = −0.662 | contralateral: *p* < 0.001; *d* = −0.717).

Also, ipsilaterally presented deviants elicited higher amplitudes than contralaterally presented deviants in the frontal-cluster (*p* = 0.009; *d* = −0.359). In particular, contralaterally presented deviants caused higher amplitudes in the left- (*p* < 0.001; *d* = 0.439) and right-cluster (*p* < 0.001; *d* = 0.515) than ipsilaterally presented deviants. The three-way-interaction between laterality, stimuli and body part was not significant.

#### N140 Latency

**Figure 5:**
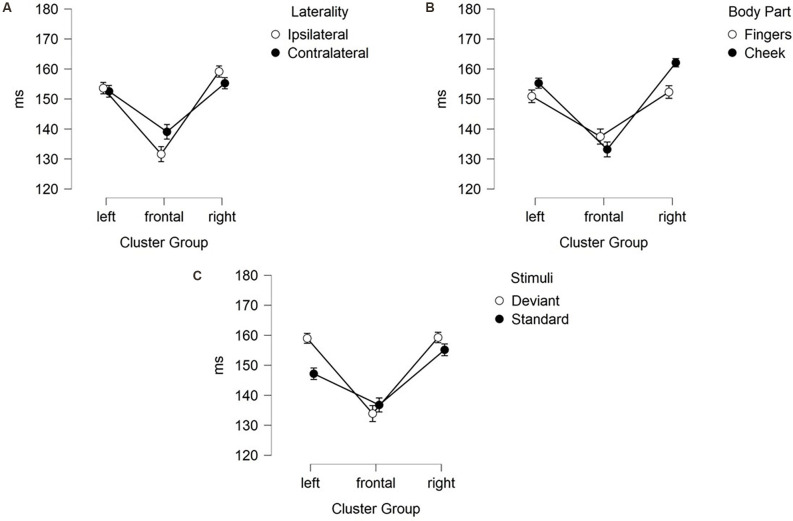
Mean values (ms) of N140 latencies with SE. Two-way-interactions: **(A)** Cluster Group x Laterality, **(B)** Cluster Group x Body Part, and **(C)** Cluster Group x Stimuli.

**Figure 6:**
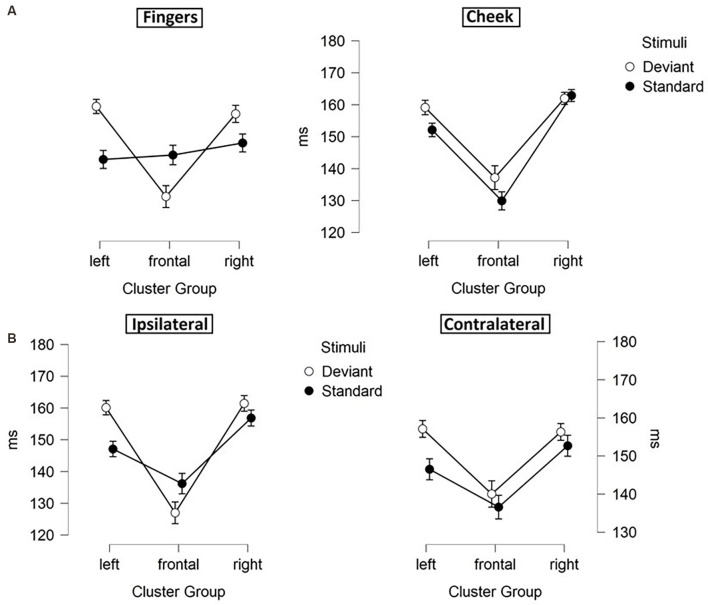
Mean values (ms) of N140 latencies with SE. Three-way-interactions: **(A)** Cluster Group x Body Part x Stimuli, **(B)** Cluster Group x Laterality x Stimuli.

The results of the latency for frontal N140 amplitudes are illustrated in Figures 5, 6 and means are detailed in Table 3. They showed significant main-effects for stimuli (*F*_(1, 21)_ = 6.956; *p* = 0.015; *η*^2^ = 0.249), body part (*F*_(1, 21)_ = 5.098; *p* = 0.035; *η*^2^ = 0.195), and cluster group (*F*_(1.59, 33.35)_ = 26.352; *p* < 0.001; *η*^2^ = 0.557). Also, a significant two-way interaction between laterality and cluster group (*F*_(1.94, 40.73)_ = 6.099; *p* = 0.005; *η*^2^ = 0.225, Figure 5A) occurred. Ipsilateral stimulation caused significantly shorter latencies in the frontal-cluster than contralateral stimulation (*p* = 0.026; *d* = −0.415). Moreover, the interaction between cluster group and body part (*F*_(1.67, 35.02)_ = 8.840; *p* = 0.001; *η*^2^ = 0.296) arose, because stimuli presented at the fingers elicited shorter latencies in the lateral right-cluster (*p* = 0.002; *d* = −0.503) than stimuli presented at the cheek (Figure 5B). Also, stimuli presented at the cheek elicited shorter latencies in the frontal-cluster than in the lateral left- (*p* < 0.001; *d* = 0.758) and right-cluster (*p* < 0.001; *d* = −0.993). A further two-way-interaction between cluster group and stimuli (*F*_(1.52, 31.99)_ = 3.937; *p* = 0.040; *η*^2^ = 0.158) was found due to the fact that deviant stimuli elicited shorter latencies in the frontal-cluster than in the lateral left- (*p* < 0, 001;* d* = 0.671) and lateral right-cluster (*p* < 0, 001;* d* = −0.678). Moreover, standard stimuli elicited shorter latencies in the left-cluster (*p* = 0.016; *d* = 0.439) than deviant stimuli (Figure 5C).

**Table 3:** Mean latencies of N140 with SE values.

Laterality of deviant	Body part	Stimuli	Cluster group	Mean (ms)	SE
Ipsilateral	Fingers	Deviant	left	162	± 3.36
			frontal	125	± 4.49
			right	160	± 3.71
		Standard	left	142	± 3.99
			frontal	143	± 4.70
			right	152	± 3.70
	Cheek	Deviant	left	158	± 3.30
			frontal	129	± 5.44
			right	163	± 3.07
		Standard	left	152	± 2.62
			frontal	130	± 4.57
			right	162	± 2.62
Contralateral	Fingers	Deviant	left	157	± 3.35
			frontal	137	± 4.74
			right	153	± 3.45
		Standard	left	143	± 4.82
			frontal	145	± 4.76
			right	143	± 3.53
	Cheek	Deviant	left	159	± 3.13
			frontal	145	± 5.43
			right	161	± 2.72
		Standard	left	151	± 2.94
			frontal	129	± 4.26
			right	163	± 2.33

Moreover, a three-way-interaction between cluster group, body part and stimuli (*F*_(1.43, 30.04)_ = 11.171; *p* < 0.001; *η*^2^ = 0.347) was found (Figure 6A). Deviants presented at the cheek elicited shorter latencies in frontal-cluster than in left- (*p* < 0.001; *d* = 0.393) and right-cluster (*p* < 0.001; *d* = −0.444). Also, deviants presented at the fingers caused shorter latencies in frontal-cluster than in left- (*p* < 0.001; *d* = 0.505) and right-cluster (*p* < 0.001; *d* = −0.463). Another three-way-interaction between laterality, cluster group and stimuli (*F*_(1.89, 39.60)_ = 5.232; *p* = 0.011; *η*^2^ = 0.199) occurred (Figure 6B). For standard stimuli, presented ipsi- as well as contralaterally, latencies were shorter in frontal-cluster than in right-cluster (ipsilateral: *p* = 0.001; *d* = −0.403 | contralateral: *p* = 0.049; *d* = −0.314). Also, ipsilateral presented deviants elicited shorter latencies than contralateral presented deviants in the frontal-cluster (*p* < 0.001; *d* = −0.445).

### P300 Component (250–550 ms)

#### P300 Amplitude

**Figure 7:**
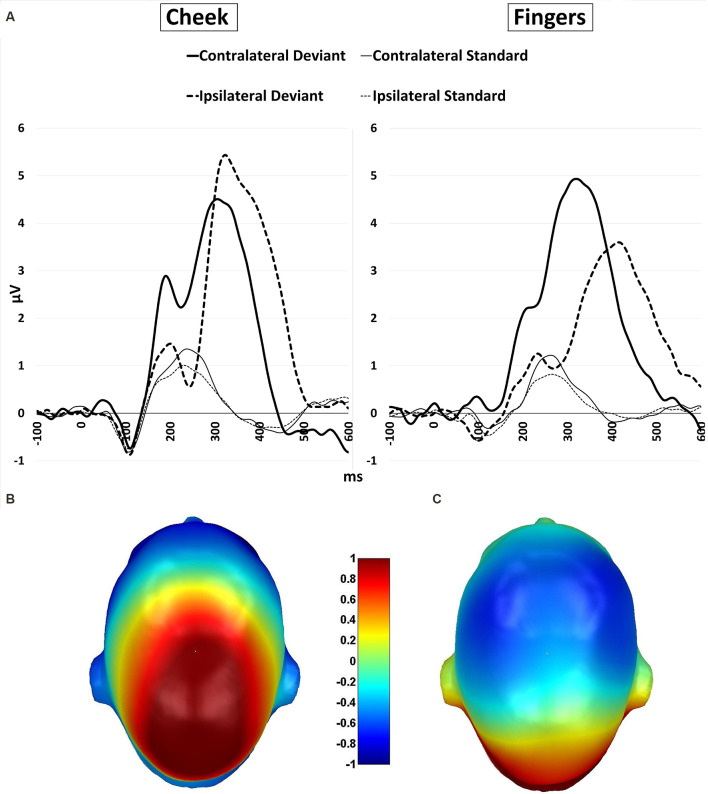
**(A)** P300 waveforms. Grand-averaged ERP waveforms for central-cluster at the cheek (left) and fingers (right) in response to contralateral presented deviant stimuli (bold black line), ipsilateral presented deviants (bold black dotted), standard stimuli in the contralateral deviant condition (thin black line) and standard stimuli presented in the ipsilateral deviant condition (thin black dotted); **(B)** topographic difference plot (ipsilateral deviant cheek − contralateral deviant cheek), **(C)** topographic difference plot (ipsilateral deviant fingers − contralateral deviant fingers). ERP, Event-Related Potentials.

For central P300 amplitudes, amplitude results are illustrated in Figure 7 and means are detailed in Table 4. They showed a significant main-effect for stimuli (*F*_(1, 21)_ = 116.360; *p* < 0.001; *η*^2^ = 0.847) and body part (*F*_(1, 21)_ = 6.361; *p* = 0.020; *η*^2^ = 0.232).

**Table 4:** Mean amplitudes of P300 with SE values.

Laterality of deviant	Body part	Stimuli	Mean (μV)	SE
Ipsilateral	Fingers	Deviant	4.36	± 0.36
		Standard	1.13	± 0.14
	Cheek	Deviant	6.57	± 0.61
		Standard	1.18	± 0.14
Contralateral	Fingers	Deviant	5.71	± 0.47
		Standard	1.50	± 0.14
	Cheek	Deviant	5.34	± 0.59
		Standard	1.50	± 0.14

Furthermore, a significant two-way interaction between laterality and body part (*F*_(1, 21)_ = 18.185; *p* < 0.001; *η*^2^ = 0.464) was found. *Post hoc* *t*-tests showed significantly higher amplitudes for ipsilateral stimulation at the cheek than ipsilateral stimulation at the fingers (*p* < 0.001; *d* = −0.723), contralateral stimulation at the fingers (*p* = 0.002; *d* = −0.601) and contralateral stimulation at the cheeks (*p* = 0.049; *d* = −0.431). There was no significant difference between the contralateral stimulation at the fingers and contralateral stimulation at the cheek.

A further two-way-interaction between body part and stimuli (*F*_(1, 21)_ = 4.959; *p* = 0.037; *η*^2^ = 0.191) was due to the fact that deviants presented at the cheeks showed significantly higher amplitudes than deviants presented at the fingers (*p* = 0.010; *d* = −0.512). Crucially, a three-way interaction between laterality, body part and stimuli was found (*F*_(1, 21)_ = 18.916; *p* < 0.001; *η*^2^ = 0.474; Figure 7). Ipsilateral deviant stimulation at the cheeks caused higher P300 responses than ipsilateral deviant stimulation at the fingers (*p* < 0.001; *d* = −0.714) and contralateral deviant stimulation at the cheeks (*p* = 0.003; *d* = 0.444). Also, contralateral presented deviants at the fingers caused higher amplitudes than ipsilateral presented deviants at the fingers (*p* < 0.001; *d* = −0.488). There was no significant difference between the contralateral deviant stimulation at the fingers and cheeks.

#### P300 Latency

**Figure 8:**
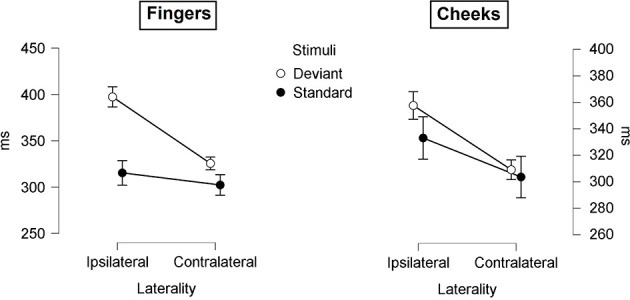
Mean values (ms) of P300 latencies with SE. Three-way-Interaction: Laterality x Body part x Stimuli.

The mean latencies for central P300 amplitudes are summarized in Table 5. Results showed significant main-effects for stimuli (*F*_(1, 21)_ = 6.509; *p* = 0.019; *η*^2^ = 0.237) and laterality (*F*_(1, 21)_ = 45.686; *p* < 0.001; *η*^2^ = 0.685). Further, a significant two-way interaction between laterality and stimuli (*F*_(1, 21)_ = 9.157; *p* = 0.006; *η*^2^ = 0.304) indicated shorter latency for contralateral deviants than for ipsilateral deviants (*p* < 0.001; *d* = 1.044). Critically, a three-way interaction between laterality, body part and stimuli was found (*F*_(1, 21)_ = 4.488; *p* = 0.046; *η*^2^ = 0.176; Figure 8). Contralateral deviants presented at the cheeks showed significantly shorter latency than ipsilaterally presented deviants at the cheeks (*p* = 0.002; *d* = 0.469) and ipsilaterally presented deviants at the fingers (*p* < 0.001; *d* = 0.641). Also, contralateral deviants at the fingers showed significantly shorter latencies than ipsilateral presented deviants at the fingers (*p* < 0.001; *d* = 0.693).

**Table 5:** Mean latencies of P300 with SE values.

Laterality of deviant	Body part	Stimuli	Mean (ms)	SE
Ipsilateral	Fingers	Deviant	397	± 9.47
		Standard	315	± 16.48
	Cheek	Deviant	357	± 7.63
		Standard	333	± 18.42
Contralateral	Fingers	Deviant	326	± 6.20
		Standard	302	± 13.88
	Cheek	Deviant	309	± 5.02
		Standard	303	± 15.15

## Discussion

We investigated, to what extent patterns of N140 and P300 responses in a somatosensory oddball paradigm differ between finger and face stimulation when using ipsilateral and contralateral intensity deviants covering bigger or smaller patches of skin. For the N140 component, we found significant interactions regarding laterality, stimuli, and cluster group in the analysis of amplitudes as well as latencies. We found higher responses for contralateral presented deviants than for ipsilateral presented deviants despite spatially more extended ipsilateral deviants but with no significant differences between body parts. For the P300 deflection, we found similar results but only for the stimulation at the fingers with higher amplitudes and shorter latencies in the contralateral stimulation. For the face stimulation, ipsilateral deviants elicited higher amplitudes than contralateral deviants, corresponding to the larger spatial extend of the ipsilateral deviant.

### N140

Our results showed, that N140 responses were significantly higher and latencies significantly shorter in the fronto-central-cluster than in the temporal left- and right-cluster reflecting N140 topography.

Also, stimulation at the cheek caused higher N140 amplitudes but significantly longer latencies than the stimulation at the fingers regardless of the laterality of stimulus presentation. Higher amplitudes may be attributed to the fact that the face has a larger representation than the fingers in the somatosensory homunculus (Penfield and Rasmussen, 1950). Longer latencies for the stimulation at the cheek could be reduced to the tactile threshold of the body part and task difficulty. It is known that the cheek has a much higher threshold than the fingers (Weinstein, 1968; James et al., 2000; Haggard et al., 2003) and that the N140 component is affected by task difficulty which is reflected in longer latencies (Michie et al., 1987). Also, results showed significantly shorter latencies for ipsilateral deviant stimuli in the frontal-cluster than for contralateral deviant stimuli which can be reduced to the spatial extent of the deviant in ipsilateral stimulation. Nakajima and Imamura (2000a) showed that the peak latency of the N140 component decreases with increasing stimuli intensity due to synchronously more activated connections and neurons. Another finding was that the contralateral deviant stimulation elicited more negativity than ipsilateral deviant stimulation, despite the ipsilateral deviant covering a bigger area. Shen et al. (2018) reported significantly higher amplitudes in early automatic somatosensory mismatch response when the distance between the cortical representations of stimulating locations is larger. The present study aimed to extend this to the N140 response in an active oddball paradigm. Indeed, contralateral deviants elicited higher amplitudes than ipsilateral ones, which could be accounted for by stronger intra-hemispheric interference elicited by ipsilateral deviants. Since there was no further interaction with a body part, our results suggest that across hemispheres, the distance between body part representations does not significantly affect N140 amplitudes. Moreover, for contralateral deviant stimulation, the difference between standard and deviant was restricted to the temporal channel groups, whereas for ipsilateral stimulation, it was more widely distributed across fronto-central and temporal sites.

Our hypothesis that the somatosensory stimulation at body parts with different degrees of separation between the somatotopic representations (fingers vs. face) causes significant differences in the N140 response was not confirmed. We could show that the laterality of stimulus presentation has an effect on the N140 component, contralateral deviant stimulation leading to higher N140 amplitudes than ipsilateral deviant stimulation, but we observed no significant differences between stimulated body parts. Shen et al. (2018) showed that, at least within one hemisphere, stimulating body parts with a larger distance between the cortical representations in the somatosensory cortex leads to higher MMN deflections. In our study, the interhemispheric distance between the cortical representation between fingers and cheeks appears to have no effect on the N140 response, as otherwise cheeks and fingers should have responded differently to the contralateral stimulation. Nevertheless, we could show that the early N140 ERP is affected by intra-hemispheric interference: Despite the ipsilateral deviant covering more skin surface, amplitudes were larger for the contralateral stimulation. This finding is consistent with the results of multiple other studies. Pang and Mueller (2015) and Severens et al. (2010) showed only intra-hemispheric interference, with significantly smaller SSSEPs for the within-hand condition vs. the between-hand stimulation although events had longer duration in the within-hand paradigm. We have extended this finding to stimulation at the cheek, suggesting it to be a rather general phenomenon in early somatosensory processing.

### P300

For the P300 component results showed, consistent with our hypothesis, significantly higher P300 responses and shorter latencies in the contralateral stimulation at the fingers than in the ipsilateral stimulation at the fingers although ipsilateral deviants covered a bigger area. This finding extends former SSSEP studies like Pang and Mueller (2015) and Severens et al. (2010) who explored within-hand stimulation. Our study could show that also the P300 is affected by intra-hemispheric interference in somatosensory stimulation when adjacent fingers are stimulated. An explanation may be lateral feedback inhibition. For the localization of the exact stimulation-spot in multiple-area-stimulation with neighboring representation, excitatory and inhibitory synaptic connections may overlap and interfere (Biermann et al., 1998). Interestingly, this interference was not observed in the stimulation at the cheek. We hypothesize this to be due to a single representation of the face in the somatosensory cortex (Penfield and Rasmussen, 1950). In contrast, ipsilateral stimulation at the cheek caused significantly higher amplitudes than ipsilateral stimulation at the fingers.

Furthermore, as an effect caused by the larger area subtended by ipsilateral deviants, our results showed, also consistent with our hypothesis, that the ipsilateral stimulation at the cheek causes a higher P300 response than the contralateral stimulation at the cheek. This reflects the finding of Nakajima and Imamura (2000a) that the P300 deflection increases with increasing stimulus intensity and also with higher distinguishability of the used stimuli (Linden, 2005). But this finding cannot only be reduced to the exogenous component, stimulated skin area, but has to involve also to an endogenous psychological component. P300 generators not only receive input from the somatosensory system but also from higher cerebral centers (Nakajima and Imamura, 2000a) Moreover, cognitive processes like attention and working memory are involved in the generation of the P300 potential (Linden, 2005). Descriptively consistent with our hypothesis, our results showed higher amplitudes in the contralateral stimulation at the hand than the contralateral stimulation at the cheeks, but this finding was not significant. With regard to P300 latencies: Albeit not significant, contralateral deviants presented at the cheeks tended to show shorter latencies than contralateral deviants at the fingers. Shen et al. (2018) could show similar findings. Shorter latencies for the neck/lip stimulation in contrast to lip/hand stimulation, but this finding was not further discussed by the authors. Maybe this finding can be attributed to the sensory pathway of the stimulated area to the somatosensory system, which is shorter for the cheeks than for the fingers resulting in a longer neural processing time for stimulating the fingers. Kodama et al. (2016) showed that P300 peak latency increases with longer sensory pathways (e.g., arm vs. leg).

In conclusion, we could show that ipsilateral stimulation causes non-selective intra-hemispheric interference in the early N140 in that there was no difference between the stimulation at the fingers and cheeks. For the P300, however, fingers and cheeks differed and interference seemed to occur only at the fingers, not at the cheek, resulting in larger P300 amplitudes and shorter latencies following stimulation at the cheek. Within the hemisphere, Shen et al. (2018) reported significantly higher amplitudes in early automatic somatosensory mismatch response with increasing distance between the cortical representations of stimulated locations. When increasing the distance between cortical representations across the hemispheres, we could not confirm this finding regarding the N140 component. Since the MMN and the N140 are distinct components, the present pattern may not extend to the MMN when stimulating across the hemispheres. This could be tested in further studies.

For the P300 intra-hemispheric interference was restricted to the stimulation at the fingers resulting in higher responses and shorter latencies for the contralateral stimulation at the fingers than the ipsilateral stimulation at the fingers despite larger area ipsilateral deviants and higher amplitudes for the ipsilateral stimulation at the cheeks than contralateral stimulation at the cheeks. This interference may be reduced by increasing the cortical distance of stimulated areas (Mountcastle, 1984), or like in our study, by stimulating areas with a larger or uniform representation in the somatosensory cortex. Regarding stimulated body locations, Shen et al. (2018) revealed that, at least within one hemisphere, the P300 is affected by the distance between these body parts on the body surface. Our results showed, at least descriptively, higher responses in the contralateral stimulation at the fingers than at the cheeks, but this finding was not significant. Together with the similar finding for the N140, this suggests that mechanisms of intra-hemispheric stimulation differ from inter-hemispheric ones.

### Limitations and Conclusion

Replication of our study is desirable with extension to other combinations of body parts to test if the present effects are caused by body parts with a single representation or bigger representation in the somatosensory cortex, like the face (Penfield and Rasmussen, 1950), than body parts with overlapping or neighboring cortical representations. In response to a reviewer’s helpful suggestion, we checked if gender has an effect on our data. Because only five males took part in this study, we cannot tell, whether observed effects would be replicable, but N140 amplitudes in response to deviant stimuli were overall more negative for females than for males (Gender X Stimuli (*F*_(1, 20)_ = 5.752; *p* = 0.026; *η*^2^ = 0.223); *M*_diff_= −0.707; *p* = 0.020; *d* = −0.613). For the P300, responses to stimuli presented at the cheeks were significantly higher in female than male subjects (Body Part X Gender (*F*_(1, 20)_ = 9.644; *p* = 0.006; *η*^2^ = 0.325); *M*_diff_= 1.968; *p* = 0.012; *d* = 0.681). Our sample had a similar gender distribution to other recent ones in the area and we are not aware of any previous reports on gender differences in somatosensory ERPs. Still, a replication of our study should be balanced for gender and a power calculation should determine sufficient power for interpretation of between group effects.

In the present data stimulation at the cheeks caused significantly higher responses than the stimulation at the fingers. Jin et al. (2020) developed a novel brain-computer-interface (BCI) paradigm in which target tactile stimuli are presented at participant’s cheeks. Their results showed a better single-trial classification when stimulating the cheeks instead of the wrists, suggesting that the stimulation at the cheek might be useful for BCI. Like Jin et al. (2020), we could show that the cheeks could be considered as a good alternative to hand stimulation due to higher responses in the N140 and P300 components. This knowledge can inform the design of somatosensory stimulation-based brain-computer interfaces, particularly for individuals who cannot control their eye gaze or paraplegic patients. There are numerous BCIs using particularly visual but also auditory stimuli to elicit brain signals (Farewell and Donchin, 1988; Hoffmann et al., 2008; Nijboer et al., 2008; Pires et al., 2008; Salvaris and Sepulveda, 2009; Kim et al., 2012; Sugi et al., 2018), but somatosensory stimulation for BCIs is a feasible alternative to auditory and visual ones (Brouwer and Erp, 2010; Van der Waal et al., 2012 ; Kaufmann et al., 2013) especially when deviant stimuli are presented contralaterally.

## Data Availability Statement

The data that support the findings of this study are available from the corresponding author upon reasonable request.

## Ethics Statement

The studies involving human participants were reviewed and approved by Ethik-Kommission der Universität Bielefeld Postfach 100131, D-33501 Bielefeld. Written informed consent for participation was not required for this study in accordance with the national legislation and the institutional requirements.

## Author Contributions

LL and JK contributed to the conception and design of the study. LL organized the database, performed the statistical analysis, and wrote the manuscript. SZ, JA, and TH contributed to materialize the stimulation system, the haptic devices, and firmware programming. All authors contributed to the article and approved the submitted version.

## Conflict of Interest

The authors declare that the research was conducted in the absence of any commercial or financial relationships that could be construed as a potential conflict of interest.

## Publisher’s Note

All claims expressed in this article are solely those of the authors and do not necessarily represent those of their affiliated organizations, or those of the publisher, the editors and the reviewers. Any product that may be evaluated in this article, or claim that may be made by its manufacturer, is not guaranteed or endorsed by the publisher.

## Footnotes

^1^ http://www.biosemi.com
